# Structural nodal efficiency in executive-function-related regions moderates individual and paired-player creativity

**DOI:** 10.3389/fpsyg.2026.1781779

**Published:** 2026-05-26

**Authors:** Ching-Lin Wu

**Affiliations:** 1 Program of Learning Sciences, National Taiwan Normal University, Taipei, Taiwan; 2 Institute for Research Excellence in Learning Sciences, National Taiwan Normal University, Taipei, Taiwan

**Keywords:** collaborative performance, connectome, creativity, diffusion tensor imaging, remote associates test

## Abstract

This study investigates how the structural nodal efficiency of selected executive-function-related regions moderates the relationship between individual and collaborative performance on divergent thinking tasks and Chinese Radical Remote Associates Tests (CRRAT). Participants completed the Alternative Uses Task and CRRAT in individual and paired-player modes, while undergoing diffusion-based structural brain imaging. Results revealed that the nodal efficiency of selected nodes located within the superior frontal, inferior frontal, anterior cingulate, and posterior cingulate regions significantly moderated the relationship between individual and collaborative creativity performance. When node efficiency in these regions was higher, individual performance positively predicted collaborative performance. Additionally, the nodal efficiency of selected nodes located within the superior and middle temporal regions moderated the relationship between individual and paired performances on the CRRAT. In these regions, lower node efficiency was associated with a stronger positive predictive relationship between individual and collaborative CRRAT performance. These findings highlight the moderating effects of regional structural network efficiency in creativity, offering neural insights into individual and collaborative cognitive performance in one-on-one interactive contexts.

## Introduction

### Creative problem-solving during interactive situations

Creative problem-solving can take open-ended and closed forms and includes both divergent thinking and insight-based problem solving (Javaid and Pandarakalam, 2021). Firstly, divergent thinking requires individuals to generate multiple responses to problems that do not have a single standard answer. Divergent thinking is commonly assessed in terms of fluency, flexibility, originality, and elaboration (Kenett and Faust, 2019). Thus, divergent thinking reflects the diversity and originality of ideas generated during free association. It is commonly measured through Alternative Uses Tasks (AUT) (Torrance, 1974). Conversely, insight problem solving requires individuals to use limited cues to converge on a single correct answer. This process often involves restructuring the initial problem representation and overcoming impasses, sometimes accompanied by an “aha” experience. Insight problem solving is commonly assessed using the Remote Associates Test (RAT), which requires participants to identify a single response that links several seemingly unrelated cues (Bowden and Jung-Beeman, 2003; Huang et al., 2019). In the Chinese version of the RAT, insight problem solving can be assessed using the Chinese Radical Remote Associates Test (CRRAT), which has shown adequate validity as a measure related to insight-based problem solving (Chang et al., 2016; Wu, 2019).

Creative problem-solving may differ between collaborative and individual contexts (Wu, 2022). Early research showed that collaborating with a more capable partner could improve the originality of highly creative participants’ responses (Michinov et al., 2015). In a previous study using the online creativity paradigm, lower-performing individuals improved more in paired-player mode, and more frequent reference to another participant’s responses was associated with better performance in fluency, flexibility, and CRRAT accuracy (Wu, 2022). Taken together, these findings suggest that interactive contexts may enhance creative performance by allowing individuals to incorporate others’ ideas. Taken together, these findings suggest that interactive contexts may enhance creative performance by allowing individuals to incorporate others’ ideas (Yalçın and Erden, 2021). In summary, collaborative creativity appears to depend partly on how individuals integrate their own knowledge with external input.

In addition to interpersonal influence, task context may also shape creativity in interactive situations. Wu and Chen (2022) found that individuals performed better in paired-player than in single-player mode, and that cooperative contexts were especially beneficial for CRRAT performance. These findings suggest that individual characteristics may also contribute to creativity in interactive contexts.

### Creativity, executive functions, and the brain connectome

Executive function is a multidimensional construct that includes shifting, working memory, and inhibition (Diamond, 2013; Miyake et al., 2000). Creativity is also multidimensional, and its relation to these executive components may vary across forms of creative problem solving (Ellis et al., 2020). Shifting, working memory, and inhibition refer, respectively, to flexible perspective change, temporary maintenance and use of information, and the suppression of dominant but task-irrelevant responses.

Executive functions are closely related to creativity, but their associations may vary across creative tasks (Ellis et al., 2020). Shifting is generally more relevant to divergent thinking because it supports movement across semantic categories and the generation of multiple ideas. By contrast, remote associates tasks rely more strongly on working memory and controlled retrieval because participants must maintain multiple cues and evaluate candidate answers (Chein and Weisberg, 2014; Lee et al., 2014). Inhibitory control may also support divergent thinking by helping individuals suppress dominant but unoriginal responses (Radel et al., 2015; Khalil et al., 2019). Prior research indicates that working memory supports both divergent thinking and RAT/CRRAT performance, particularly by facilitating the retention and comparison of task-relevant information (Chein and Weisberg, 2014; Lee et al., 2014; Zabelina et al., 2019). Inhibition has been associated with better fluency and originality in divergent thinking, but not consistently with RAT performance (Khalil et al., 2019; Radel et al., 2015).

Overall, divergent thinking appears to involve shifting, working memory, and inhibition, whereas RAT/CRRAT performance appears to depend more strongly on working memory. Rather than attempting to represent the full executive-function network, the present study focused on six *a priori* candidate regions that have been linked in prior research to executive processing and creativity-related cognition: the superior frontal gyrus (SFG), inferior frontal gyrus (IFG), superior temporal gyrus (STG), middle temporal gyrus (MTG), anterior cingulate cortex (ACC), and posterior cingulate cortex (PCC). These regions were selected because previous research has linked them to cognitive flexibility, inhibitory control, working memory, controlled retrieval, and creative idea generation. At the same time, this set of regions should not be interpreted as an exhaustive representation of the broader executive-function system, which may also involve additional cortical areas, including parietal regions.

Previous studies have linked the ACC and PCC to inhibition (El Haj, 2016; Funch Uhre et al., 2022; Mansouri et al., 2009; Mattavelli et al., 2024; Wang et al., 2025; Zhao et al., 2023) and working memory (Kobayashi et al., 2021; Seamans and Floresco, 2022), the frontal regions to cognitive flexibility (Dajani and Uddin, 2015; Swainson et al., 2003; Tian et al., 2025; Wang et al., 2021), working memory (Banich et al., 2023; Chen et al., 2022; Vartanian et al., 2013), and inhibition (Cassotti et al., 2016; Hu et al., 2016; Gavazzi et al., 2023; Koechlin, 2016; Mattavelli et al., 2024; Morie and Potenza, 2021), and the temporal regions to verbal working memory (Gurrieri et al., 2025; Roger et al., 2022; Saldarini et al., 2022; Wang et al., 2025) and controlled semantic processing (Wu et al., 2020). Taken together, these findings provide a theoretical basis for examining whether the structural efficiency of these selected regions moderate creativity performance in individual and interactive contexts.

Beyond examining isolated regions, it is also important to consider how they are embedded within the broader structural connectome. Graph-theoretical analysis characterizes the white-matter network of the brain by estimating how efficiently information can be transferred across interconnected regions (Bullmore and Sporns, 2009). Graph-theoretical metrics can be used to estimate both global and local properties of information transfer within the structural network. For example, individuals with eminent creative achievement have been found to show distinct large-scale network properties relative to non-eminent controls (Anderson et al., 2024). In a previous study by the present author, whole-network topological properties moderated the association between single-player and paired-player originality, but not fluency, flexibility, or CRRAT performance (Wu, 2024).

### The present study

The present study examined whether the nodal efficiency of six selected executive-function-related regions moderates the association between single-player and paired-player creativity performance. This study builds on prior work on interactive creativity, including research on internal processes (Wu, 2022), task context (Wu and Chen, 2022), DMN connectivity (Wu, 2023), and whole-network topology (Wu, 2024). Importantly, the present study used an independent sample, and there was no participant overlap with the samples reported in Wu (2023) or Wu (2024). Whereas Wu (2023) focused on DMN connectivity and Wu (2024) on whole-network topology, the present study focused on the nodal efficiency of six selected regions. Thus, the present manuscript differs from Wu (2024) in both sample and level of analysis. Specifically, performance in the single-player mode on divergent thinking (fluency, flexibility, and originality) and the Chinese Radical Remote Associates Test (CRRAT) was used to predict performance in the paired-player mode on the same tasks, with the nodal efficiency of the six selected regions entered as moderators while controlling for age and sex. This region-specific approach was intended to clarify how executive-function-related nodal efficiency contributes to creativity in one-on-one interactive contexts.

Previous research has shown that shifting, inhibition, and working memory are positively associated with divergent thinking, whereas working memory is also significantly related to CRRAT performance (Chein and Weisberg, 2014; Ellis et al., 2020; Lee et al., 2014; Zabelina et al., 2019). Neural evidence further indicates that frontal and cingulate regions are involved in executive-function processes, while superior and middle temporal regions are more closely linked to working memory (Cassotti et al., 2016; Mattavelli et al., 2024; Roger et al., 2022; Saldarini et al., 2022; Zhao et al., 2023). In addition, graph-theoretical studies suggest that network efficiency predicts divergent thinking performance in both single- and paired-player modes (Wu, 2024). Accordingly, the present study hypothesized that the nodal efficiency of frontal and cingulate regions would moderate divergent thinking across individual and paired-player contexts, whereas the nodal efficiency of superior and middle temporal regions would moderate CRRAT performance.

## Method

### Participants

A total of 135 neurologically normal adults (60 males, 75 females), with ages ranging from 20 to 30 years (Mean = 22.59, SD = 2.19). All participants were native speakers of traditional Chinese and exhibited no significant barriers to their Chinese reading abilities. This study has been approved by the Institutional Review Board of National Taiwan Normal University. All participants began the study after understanding the research content and signing the informed consent form.

### Behavioral measures of individual and paired-player creativity

The participants were asked to answer an online creativity task. The details of which are discussed below.

#### Online interactive creativity task platform

**Figure 1:**
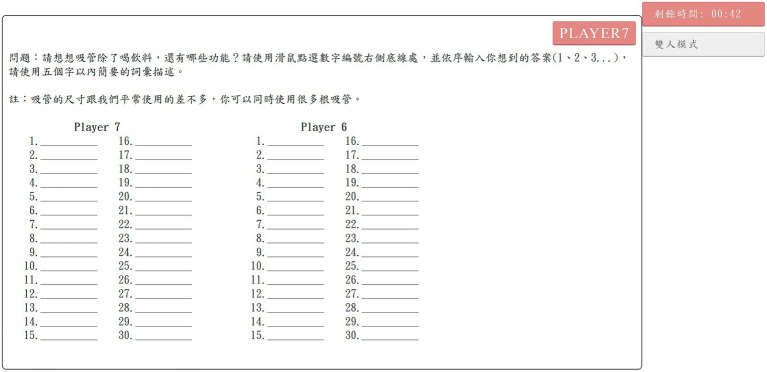
Surface of the online interactive creativity task platform. 「問題:請想想吸管除了喝飲料還有哪些功能?請用滑鼠點選數字編號右側底線處並依序輸入你想到的答案(1、2、3.)請使用五個字以內簡要的詞彙描述。」Question: Please consider alternative functions that straws can serve beyond drinking beverages. Click on the bottom line to the right of each number and enter your ideas sequentially (1, 2, 3,.). Please keep each response concise and use no more than five words. 「註:吸管的尺寸跟我們平常使用的差不多你可以同時使用很多個吸管。」Note: The straws are similar in size to standard bottles. You may also consider using multiple straws simultaneously in your responses. 「剩餘時間」-Remaining time. 「雙人模式」-Paired-player mode.

This paper developed an online creativity test on an interactive online testing platform (Wu et al., 2022), which includes two types of creativity tests: the divergent thinking test (unusual uses association) and the CRRAT (Chang et al., 2016). The online creativity test comprises sections for test questions, response presentation, response area, remaining time, and operational mode status. It allows for both individual and interactive responses. In the paired-player mode, participants could see another participant’s responses and use them as a reference while completing the creativity tests. The “another participant” was a real participant performing the same task at the same time through the online platform. Pairing between participants was randomly assigned. Importantly, participants were not informed of their partner’s age, sex, or any other personal characteristics. Thus, the paired-player condition was designed to capture the effect of exposure to another person’s task response while minimizing the influence of explicit social-category information (Figure 1).

#### Divergent thinking task (alternative uses task)

This task includes the unusual uses associations for two items: “unusual uses for a plastic bottle” and “unusual uses for a straw.” Participants’ fluency, flexibility, and originality were measured in this task. The task was automatically scored by a computer. The automated scoring program segmented each participant’s responses using the Jieba word segmentation system and compared the segmented results with established response norms to assign a category and originality score (0, 1, or 2) to each response. It then computed the total number of valid responses, distinct categories, and summed originality scores for each participant. Consistency between computer-based and manual scoring was high, with Pearson correlations (*r*) ranging from 0.92 to 0.99 across the two item sets and the three scoring indices (*r*s = 0.99, 0.92, 0.97, 0.97, 0.92, 0.95). It had good criterion-related validity with other divergent thinking tasks such as the unusual uses association for bamboo chopsticks (Wu et al., 1998) and newspapers (Hsu et al., 2012) (*r*s = 0.79, 0.54, 0.58, 0.75, 0.51, 0.60), as well as discriminant validity with the CRRAT (*r*s = 0.05, 0.10, 0.14, 0.17, 0.18, 0.18).

#### Chinese radical remote associates test (CRRAT)

This test consists of two sets of 20 items each, with items derived from the test developed by Chang et al. (2016). Each item presents three Chinese character components, such as: 「女」(nǚ, meaning “female” or “woman”), 「子」(zǐ, meaning “child” or “son”), and 「禾」(hé, meaning “grain” or “cereal”), requiring participants to think of a common valid Chinese character word that can be formed from these three components, with the answer being 「乃」(nǎi, meaning “to be” or “thus”). One point is awarded for each correct answer. This test demonstrated stable internal consistency (Cronbach’s *α* = 0.80, 0.79) and showed appropriate criterion-related validity with insight problems (*r*s = 0.48, 0.38) and the Chinese vocabulary remote associates test (*r*s = 0.58, 0.48) (Wu et al., 2022).

### Procedure

**Figure 2:**
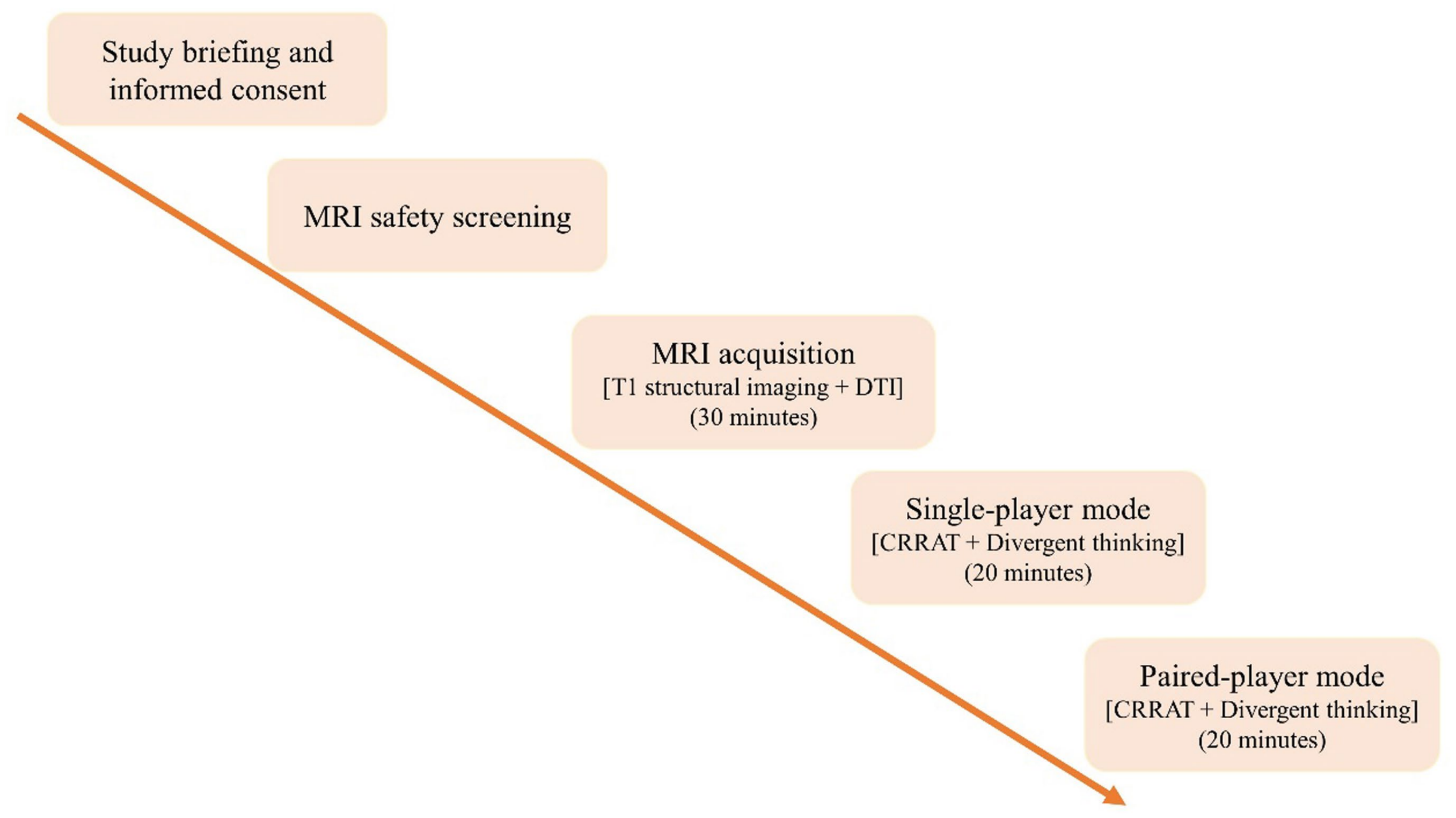
Overview of the experimental procedure. *Note.* Participants first completed informed consent and MRI safety screening, followed by structural and diffusion MRI acquisition. After scanning, they completed the CRRAT and divergent thinking task in single-player mode, followed by the same tasks in paired-player mode on the online interactive creativity platform.

Before conducting the formal study, participants were thoroughly informed about the relevant information regarding the research, including the research purpose, procedure, and participants’ rights. All participants provided one written informed consent covering both the MRI procedures and the subsequent behavioral tasks before the study began. Before scanning, participants also completed the standard MRI safety screening procedure. The scanning was divided into two phases: T1 structural imaging and diffusion tensor imaging, requiring approximately 30 min. After the scanning session, participants sequentially completed the CRRAT and the divergent thinking test in the single-player mode, followed by the CRRAT and the divergent thinking test in the paired-player mode. Each task lasted approximately 10 min, and including instructions, the total duration of the session was about 50 min. The entirety of the study lasted approximately 80 min, including MRI scanning and the implementation of behavioral scales (Figure 2).

### Magnetic resonance imaging acquisition

The MRI scans were conducted at the Taiwan Mind and Brain Imaging Center using a Siemens high-field MRI system equipped with a 32-channel head coil with a 90-degree phase difference and input or output functionality.

First, the structural imaging (T1 anatomical image) scan was performed, utilizing high-resolution T1-weighted structural scanning to localize brain activation and connectivity. The pulse sequence for the structural imaging was 3D-MPRAGE, with the following scanning parameters: TR = 2,530 ms, TE = 3.30 ms, FA = 7°, matrix size = 256 × 256 mm^2^, FOV = 256 × 256 mm^2^, slice number = 192, thickness = 1 mm, and spatial resolution of 1 × 1 × 1 mm^3^.

Subsequently, diffusion tensor imaging (DTI) was performed to observe brain network connectivity by tracking the movement of water molecules within brain fiber bundles. The scanning parameters were set as follows: TR = 11,000 ms, TE = 98 ms, Number of Directions = 64, *b*-value = 1,000 s/mm^2^, matrix size = 128 × 128 mm^2^, FOV = 224 × 224 mm^2^, slice number = 70, thickness = 1.8 mm, and spatial resolution of 1.8 × 1.8 × 1.8 mm^3^.

### Data preprocessing

The image preprocessing operations included the extraction of the skull and other non-brain materials from the images (Leemans and Jones, 2009); correction and realignment for deviations due to participant head movement and scanning distortions; fitting and local decomposition of the diffusion tensor; and calculation of specific anisotropy indicators. For atlas-based node definition, each participant’s diffusion data were registered to the corresponding anatomical image, and the anatomical image was subsequently normalized to standard MNI space. The inverse transformation was then used to project the atlas parcellation from MNI space into each participant’s native diffusion space for tractography-based network construction. This procedure ensured anatomical correspondence between the diffusion data and the atlas-defined nodes. The researchers utilized PANDA (Pipeline tool for diffusion MRI) (Cui et al., 2013) to perform all image preprocessing. This software can integrate the execution of related tools such as the FMRIB Software Library (FSL) (Smith et al., 2004), Pipeline System for Octave and Matlab (PSOM) (Bellec et al., 2012), Diffusion Toolkit (Wang et al., 2007), and MRIcron.^1^

#### Construction of binary white matter connectivity networks

The structural network was defined using a high-resolution 1,024-node anatomically guided parcellation in standard 2-mm MNI template space, rather than the original coarse-resolution AAL atlas itself (Tzourio-Mazoyer et al., 2002). In the present study, the parcellation was implemented using the file AAL_Contract_1,024_2MM_4.nii, which was obtained from the PANDA framework for diffusion MRI analysis (Cui et al., 2013). This file was used as an anatomically guided node-definition scheme for diffusion tractography-based graph analysis. The use of a high-resolution AAL-based subdivision is broadly consistent with prior connectome studies in which the AAL template was subdivided into 1,024 equal-size regions for high-resolution network construction (Liao et al., 2015; Zalesky et al., 2010). To facilitate reproducibility, we report the exact file name used in the present study, together with the node IDs and MNI coordinates of the six selected candidate regions.

A high-resolution parcellation was adopted because the present study focused on node-level structural efficiency in selected executive-function-related candidate regions, rather than on coarse region-level averages. This fine-grained node definition was considered useful for increasing sensitivity to localized variation in structural network topology. At the same time, the template matrix size is reported only to describe the image space of the parcellation file and should not be interpreted as defining the number of parcels.

### Network analysis

Guided by the *a priori* hypotheses described above, the graph-theoretical analysis focused on the nodal efficiency of six left-hemisphere candidate nodes selected because of their documented relevance to executive functioning and creativity-related processing: the superior frontal gyrus (SFG), inferior frontal gyrus (IFG), superior temporal gyrus (STG), middle temporal gyrus (MTG), anterior cingulate cortex (ACC), and posterior cingulate cortex (PCC). These regions were treated as candidate nodes of interest rather than as an exhaustive representation of the executive-function network. The analysis was restricted to the left hemisphere because the behavioral tasks were language-based, administered in Chinese, and therefore more closely related to verbal-semantic processing.

Within the 1,024-node parcellation, each of the six selected candidate regions corresponded to a single node: left middle temporal gyrus (Node 40), left superior frontal gyrus (Node 47), left inferior frontal gyrus (Node 143), left superior temporal gyrus (Node 466), left posterior cingulate cortex (Node 744), and left anterior cingulate cortex (Node 1,002). Accordingly, the nodal efficiency value for each candidate region was obtained directly from its corresponding node in the structural network. Because each candidate region was represented by a single node within the high-resolution parcellation, the anatomical labels used in this study refer to the selected node located within the broader anatomical region, rather than to the entire gyrus or cortex as a whole. To improve anatomical transparency, the node IDs and center MNI coordinates of the selected nodes are reported in Table 1.

**Table 1:** Selected candidate nodes in the 1,024-node parcellation and their anatomical locations.

Anatomical label	Hemisphere	Node ID	MNI		
			*x*	*y*	*z*
Middle temporal gyrus (MTG)	Left	40	−40	−67	17
Superior frontal gyrus (SFG)	Left	47	−3	5	56
Inferior frontal gyrus (IFG)	Left	143	−22	7	−17
Superior temporal gyrus (STG)	Left	466	−44	−55	26
Posterior cingulate cortex (PCC)	Left	744	−11	−54	19
Anterior cingulate cortex (ACC)	Left	1,002	−8	20	−7

MNI, Montreal Neurologic Institute.

Nodal efficiency was used to index the extent to which a given node could communicate efficiently with all other nodes in the whole-brain structural white-matter network. Following Achard and Bullmore (2007), the nodal efficiency of a given node was defined as the average inverse shortest path length between that node and all other nodes in the network:


$$E_{\mathrm{nodal}}\left(i\right)=\frac{1}{N-1}\underset{i\neq j\in G}{\sum }\frac{1}{L_{ij}}$$

Where L_ij_ represents the shortest path length between node i and node j, and N denotes the total number of nodes in the network. Higher nodal efficiency indicates that a given region is more efficiently integrated with the rest of the structural connectome.

### Statistical analysis

Participants’ scores for fluency, flexibility, and originality in the divergent thinking test, in both single- and paired-player modes, as well as their accuracy on the CRRAT, were calculated separately. Subsequently, a paired sample *T*-test was conducted to compare the differences in participants’ individual and interactive performances in fluency, flexibility, and originality in the divergent thinking tests and the CRRAT.

Finally, this study analyzed how the node efficiency of the superior frontal gyrus (SFG), inferior frontal gyrus (IFG), superior temporal gyrus (STG), middle temporal gyrus (MTG), anterior cingulate gyrus (ACC), and posterior cingulate gyrus (PCC) moderates the relationship between individual and interactive performances in terms of fluency, flexibility, and originality in the divergent thinking tests and the CRRAT while controlling for sex and age as covariates. The study employed the Bootstrapping Method, resampling 5,000 times to obtain 5,000 estimated values and calculate the average of the estimated moderating effects, using interval estimation to test for significance. If the moderating effect was significant, the simple slope analysis was conducted to examine whether the regression coefficients of the independent variable significantly predicted the dependent variable under conditions of having one standard deviation above or below the mean.

## Result

### Performances on creativity and Chinese radical remote associates tests

Table 2 presents participants’ scores for fluency, flexibility, and originality in divergent thinking and CRRAT pass rates, across single- and paired-player modes. In the paired-player mode, participants exhibited significantly higher scores in all measures: CRRAT (*t* = 9.19, *p* < 0.001, *d* = 0.79), fluency (*t* = 9.06, *p* < 0.001, *d* = 0.78), flexibility (*t* = 11.32, *p* < 0.001, *d* = 0.97), and originality (*t* = 2.89, *p* = 0.005, *d* = 0.25).

**Table 2:** Group difference in creativity-performance between single- and paired-player modes.

	Single-player mode		Paired-player mode		*t*(134)	95% C.I.		*d*
	Mean	SD	Mean	SD		Lower	Upper	
CRRAT	0.34	0.20	0.48	0.21	9.19^**^	−0.17	−0.11	0.79
Divergent thinking								
Fluency	14.42	6.39	18.67	6.53	9.06^**^	−5.17	−3.32	0.78
Flexibility	7.10	2.24	9.56	2.69	11.32^**^	−2.89	−2.03	0.97
Originality	13.47	7.67	15.10	6.37	2.89^**^	−2.75	−0.51	0.25

CRRAT, Chinese Radical Remote Associates Test; ^**^p < 0.01.

### Moderating effects of executive function-related brain nodes

Table 3 summarizes the moderation analysis. Node efficiencies of SFG (*B* = 5.86, *t* = 2.04, *p* = 0.043), IFG (*B* = 6.00, *t* = 3.13, *p* = 0.002), ACC (*B* = 5.72, *t* = 2.04, *p* = 0.044), and PCC (*B* = 4.76, *t* = 2.17, *p* = 0.032) significantly moderated the relationship between individual and collaborative originality in divergent thinking. Under higher node efficiency in these regions: SFG (*B* = 0.60, *t* = 6.79, *p* < 0.001), IFG (*B* = 0.67, *t* = 8.49, *p* < 0.001), ACC (*B* = 0.61, *t* = 6.52, *p* < 0.001), and PCC (*B* = 0.63, *t* = 7.19, *p* < 0.001), individual originality significantly predicted collaborative originality.

**Table 3:** Moderating effects of EF-related nodal efficiency: individual and interactive performances on divergent thinking and CRRAT.

	Divergent thinking						CRRAT	
	Fluency		Flexibility		Originality			
	*B*	*t*	*B*	*t*	*B*	*t*	*B*	*t*
Ind	−0.01	< 0.01	3.84	0.30	43.75	2.08^*^	1.48	1.99
SFG	0.67	12.78^**^	0.59	6.22^**^	0.48	9.14^**^	0.65	9.97^**^
Ind × SFG	1.95	0.73	−3.62	−0.52	5.86	2.04^*^	−5.32	−1.66
Sex	2.56	2.99^*^	0.62	1.45	2.26	2.51^*^	0.01	0.45
Age	−0.03	−0.08	−0.06	−0.95	−0.23	−0.60	< 0.01	0.68
*R*	0.68		0.51		0.65		0.67	
*R* ^2^	0.46		0.26		0.43		0.44	
*F*	37.64		8.61		19.90		22.92	
Ind	19.27	0.96	9.35	0.84	69.06	3.59^**^	0.70	1.04
IFG	0.68	12.72^**^	0.60	6.45^**^	0.55	10.76^**^	0.67	10.32^**^
Ind × IFG	1.60	0.66	2.48	0.49	6.00	3.13^**^	−4.84	−1.36
Sex	2.44	2.84^*^	0.61	1.50	2.37	2.77^*^	0.02	0.85
Age	−0.03	−0.08	−0.06	−1.01	−0.21	−0.52	< 0.01	0.66
*R*	0.68		0.52		0.69		0.65	
*R* ^2^	0.46		0.27		0.47		0.42	
*F*	37.77		9.37		32.60		21.70	
Ind	−5.21	−0.19	6.68	0.50	58.78	2.14^*^	1.00	1.09
STG	0.67	12.65^**^	0.59	6.80^**^	0.48	8.26^**^	0.69	10.73^**^
Ind × STG	0.16	0.05	−5.96	−1.02	−0.56	−0.13	−8.43	−2.27^*^
Sex	2.62	2.97^*^	0.61	1.44	2.30	2.53^*^	0.02	0.61
Age	−0.02	−0.06	−0.06	−1.04	−0.17	−0.44	< 0.01	0.77
*R*	0.68		0.52		0.65		0.66	
*R* ^2^	0.46		0.27		0.42		0.43	
*F*	38.19		10.23		19.02		23.93	
Ind	11.14	0.56	−2.83	−0.31	40.94	2.02^*^	1.23	2.11^*^
MTG	0.67	12.32^**^	0.60	6.62^**^	0.48	8.49^**^	0.66	10.40^**^
Ind × MTG	2.91	0.92	2.47	0.71	0.72	0.26	−8.84	−3.20^**^
Sex	2.42	2.86^*^	0.69	1.65	2.57	2.90^*^	0.02	0.85
Age	−0.02	−0.06	−0.06	−1.03	−0.18	−0.43	< 0.01	0.69
*R*	0.68		0.51		0.65		0.67	
*R* ^2^	0.46		0.26		0.42		0.45	
*F*	39.26		9.34		19.05		25.68	
Ind	2.69	0.12	−6.04	−0.50	54.54	2.39^*^	0.48	0.56
ACC	0.67	12.50^**^	0.60	6.38^**^	0.50	9.11^**^	0.67	9.71^**^
Ind × ACC	0.90	0.32	−0.64	−0.10	5.72	2.04^*^	−1.93	−0.41
Sex	2.55	3.01^**^	0.72	1.75	2.40	2.73^*^	0.02	0.79
Age	−0.02	−0.07	−0.05	−0.89	−0.25	−0.66	< 0.01	0.66
*R*	0.68		0.51		0.66		0.64	
*R* ^2^	0.46		0.26		0.43		0.41	
*F*	36.52		8.74		22.60		19.42	
Ind	−6.43	−0.33	−2.43	−0.24	28.44	1.39	−0.09	−0.11
PCC	0.69	12.54^**^	0.58	6.33^**^	0.53	8.97^**^	0.67	8.85^**^
Ind × PCC	3.99	1.53	−8.16	−1.50	4.76	2.17^*^	−0.89	−0.22
Sex	2.64	3.09^**^	0.71	1.74	2.41	2.66^*^	0.03	0.91
Age	−0.02	−0.07	−0.06	−0.95	−0.19	−0.51	< 0.01	0.73
*R*	0.68		0.53		0.65		0.64	
*R* ^2^	0.46		0.28		0.42		0.41	
*F*	38.68		8.82		21.27		22.85	

^*^p < 0.05, ^**^p < 0.01; Ind, single-player mode; SFG, Superior Frontal Gyrus; IFG, Inferior Frontal Gyrus; STG, Superior Temporal Gyrus; MTG, Middle Temporal Gyrus; ACC, Anterior Cingulate; PCC, Posterior Cingulate; CRRAT, Chinese Radical Remote Associates Test.

**Figure 3:**
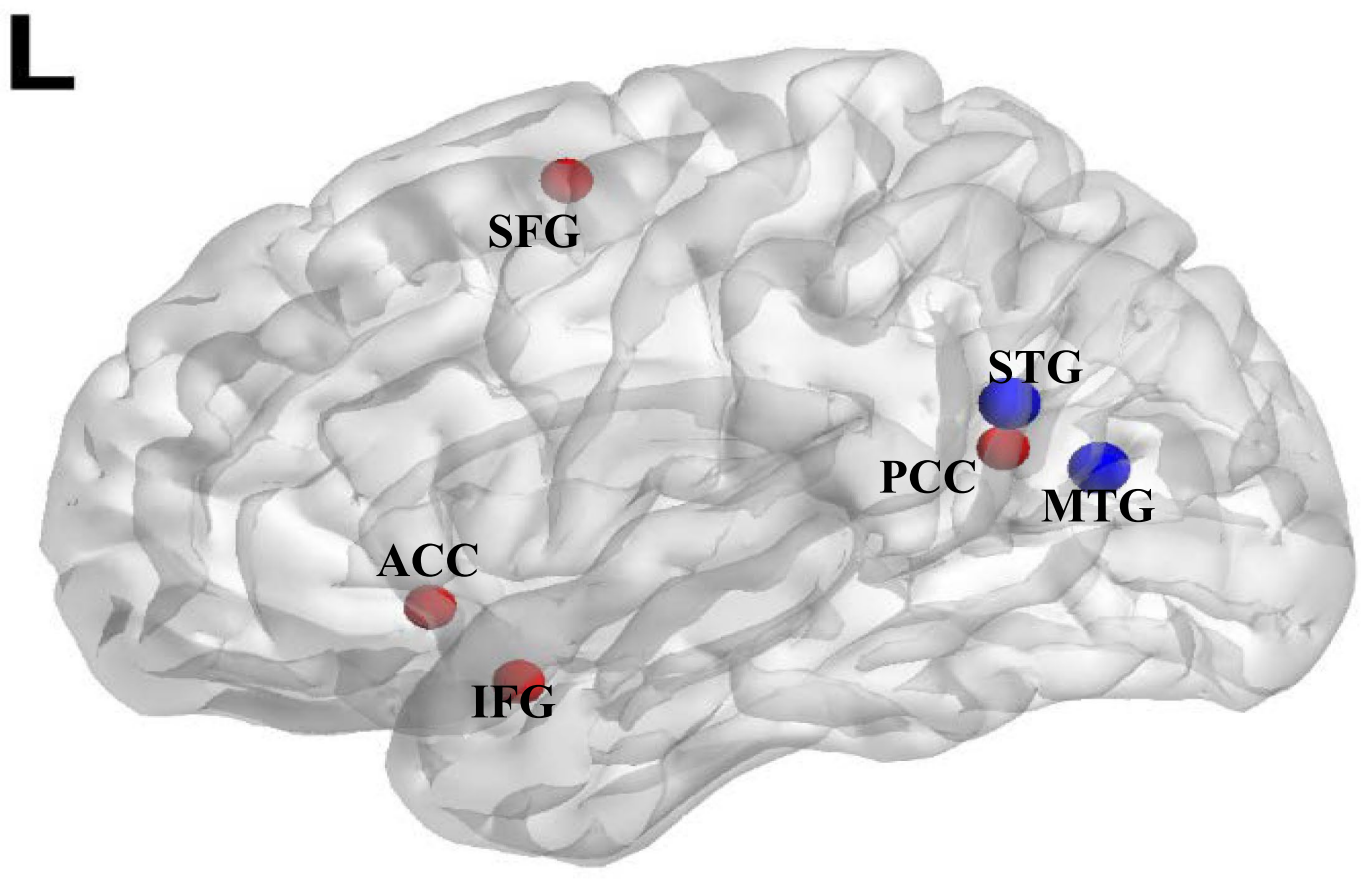
Selected executive-function-related regions in this study. *Note.* Red nodes indicate those that moderate the relationship between individual and interactive performances in divergent thinking in terms of originality; blue nodes indicate those that can moderate the relationship between individual and interactive performances on the CRRAT. The six candidate regions corresponded to the following node IDs in the 1,024-node parcellation: SFG = 47, IFG = 143, STG = 466, MTG = 40, ACC = 1,002, and PCC = 744.

Furthermore, node efficiencies of STG (*B* = −8.43, *t* = −2.27, *p* = 0.025) and MTG (*B* = −8.84, *t* = −3.20, *p* = 0.002) moderated the relationship between individual and collaborative performance on the CRRAT. Under lower node efficiency, individual CRRAT performance was a stronger predictor of collaborative performance: STG (*B* = 0.83, *t* = 8.4, *p* < 0.001) and MTG (*B* = 0.85, *t* = 10.11, *p* < 0.001). No significant moderation effects were observed for fluency or flexibility in divergent thinking. Figure 3 illustrates the executive function-related brain nodes investigated. Red nodes indicate those moderating originality in divergent thinking; blue nodes indicate those moderating CRRAT performance.

## Discussion

This study investigated how the structural nodal efficiency of selected executive-function-related candidate nodes moderated the relationship between individual and collaborative performance on divergent thinking and CRRAT tasks. By combining diffusion tensor imaging with an online creativity platform, the present study provides evidence that node-specific structural properties are associated with creativity performance in one-on-one interactive contexts.

The findings indicated that the selected frontal- and cingulate-related nodes and the selected temporal-related nodes showed different moderating effects on originality and CRRAT performance in one-on-one interactive situations. Higher nodal efficiency in the selected frontal- and cingulate-related nodes was associated with a stronger positive relation between individual and paired-player originality, whereas lower nodal efficiency in the selected temporal-related nodes was associated with a stronger positive relation between individual and paired-player CRRAT performance. Each candidate region in the present study was represented by a single node within the high-resolution parcellation. Therefore, the findings should be interpreted as reflecting the selected nodes located within broader frontal, cingulate, and temporal regions, rather than the entirety of those gyri or cortices. Future studies should examine whether similar results are observed when broader anatomical regions are represented using multiple parcels or alternative validated parcellation schemes.

Regarding node efficiency, the nodal efficiency of the selected SFG-, IFG-, ACC-, and PCC-related nodes moderated the relationship between individual and interactive performances in divergent thinking regarding originality or their ability to form novel ideas. Notably, those with higher connectivity efficiency in these regions demonstrated a stronger positive prediction of their collaborative performance based on their existing originality.

The brain regions studied in this paper are also associated with inhibition control, cognitive flexibility, and working memory (Banich et al., 2023; Chen et al., 2022; Gavazzi et al., 2023; Mansouri et al., 2009; Mattavelli et al., 2024; Tian et al., 2025), indirectly reflecting the influence of executive function operations on one’s creativity during interactive situations. Meanwhile, the IFG is also related to creativity performance (Khalil et al., 2020), and the PCC serves as a node within the default mode network (Andrews-Hanna et al., 2010) and is closely associated with creative problem-solving (Wu, 2023). This result highlights the importance of these brain nodes in contributing to individuals’ generation of creative ideas. Notably, previous studies have indicated that the functional connectivity between the ACC and the frontal lobes is related to creativity (Doss et al., 2021). From the perspective of the connectivity efficiency of these nodes and the overall network, the present study reinforces these nodes’ significant role in generating individual creativity.

Conversely, the connectivity efficiency of the STG and MTG was found to moderate the relationship between individual and interactive performances on the CRRAT. This indicates that the connections of the STG and MTG with the overall network can modulate one’s ability to effectively focus on a single answer during both independent and collaborative tasks. Particularly under conditions of lower connectivity efficiency, individuals’ existing closed creative problem-solving abilities more positively predicted their collaborative performance of the same task. These regions are associated with working memory (Roger et al., 2022; Saldarini et al., 2022), especially at the verbal level. This result suggests that lower neural transmission efficiency between brain regions involved in working memory and the overall network leads individuals to rely more heavily on their existing abilities when completing the Remote Associates Test in interactive contexts. Conversely, higher nodal efficiency may reflect a greater capacity to integrate external information relevant to working-memory demand, enabling individuals to integrate external information more effectively to solve the task collaboratively.

In addition, unlike previous research that has investigated the link between neural connectivity efficiency and creativity at the level of the whole-brain network (Anderson et al., 2024), the present study focuses on the moderating effects of connectivity efficiency at specific neural nodes across two task modes. Our findings challenge earlier conclusions suggesting that brain network connectivity efficiency does not moderate performance differences between single- and paired-player modes on the CRRAT (Wu, 2024). Instead, the results demonstrate that the efficiency of particular brain regions can indeed exert a significant influence on creative output in interactive contexts, thereby providing a more nuanced understanding of how localized neural mechanisms contribute to collaborative creativity.

Notably, this study focused on six selected executive-function-related candidate regions, each exhibiting different moderating effects on the relationship between individual and interactive performances in originality and the CRRAT. Specifically, the SFG and IFG, as well as the ACC and PCC, positively moderated one’s individual and interactive performances in originality. In contrast, the STG and MTG negatively moderated one’s individual and interactive performances on CRRAT. This indicates that six selected executive-function-related candidate regions have distinct effects during different creative problem-solving processes and indirectly demonstrates that various components of executive functions may play different roles in creativity within interactive contexts.

## Limitations and future directions

This study has several limitations. First, the analyses were limited to six selected brain regions and should not be taken as representing the full executive-function network, as other relevant regions, including parietal areas, were not examined (Horowitz-Kraus et al., 2023). Second, the study focused on nodal properties and broader structural network characteristics, but did not address functional connectivity between regions, which may provide complementary evidence regarding the neural basis of creativity in interactive contexts (Wu, 2023). Third, partner demographic information was intentionally withheld in the paired-player context to reduce explicit social expectations; however, this also limits the extent to which the findings speak to partner-related social cues. Fourth, only shifting, working memory, and inhibition were examined as representative components of executive function, leaving other relevant abilities such as categorization and vocabulary-related processes for future research. Finally, the methodological consideration is that the present study used an anatomically guided high-resolution parcellation rather than an alternative validated high-resolution framework such as the Schaefer cortical parcellation (Schaefer et al., 2018). Future studies should examine whether the present findings generalize across different validated parcellation schemes and network definitions.

## Conclusion

The present findings suggest that the structural nodal efficiency of selected executive-function-related regions moderates the association between individual and collaborative creativity performance. Higher nodal efficiency in the selected frontal- and cingulate-related nodes was related to greater originality in dyadic contexts, whereas lower nodal efficiency in the selected temporal-related nodes was associated with stronger transfer from individual to collaborative insight problem-solving. These results contribute to the literature on interactive creativity by highlighting the node-specific structural basis of creativity in one-on-one contexts.

## Data Availability

The raw data supporting the conclusions of this article will be made available by the authors, without undue reservation.

## Footnotes

1
http://www.mccauslandcenter.sc.edu/mricro/mricron/

## References

[ref9004] Achard S. Bullmore E. (2007). Efficiency and Cost of Economical Brain Functional Networks. PLOS Computat Biol. 3:e17. doi: 10.1371/journal.pcbi.0030017 PMC1794324 17274684

[ref1] Anderson A. Japardi K. Knudsen K. S. Bookheimer S. Y. Ghahremani D. G. Bilder R. M. (2024). Big-C creativity in artists and scientists is associated with more random global but less random local fMRI functional connectivity. Psychol. Aesthet. Creat. Arts 18, 550–560. doi: 10.1037/aca0000463

[ref2] Andrews-Hanna J. R. Reidler J. S. Sepulcre J. Poulin R. Buckner R. L. (2010). Functional-anatomic fractionation of the brain's default network. Neuron 65, 550–562. doi: 10.1016/j.neuron.2010.02.005, 20188659 PMC2848443

[ref3] Banich M. T. Wang K. Kim H. Leopold D. R. Reineberg A. E. Thompson L. A. . (2023). The influence of executive processing on reading comprehension during adolescence. Mind Brain Educ. 17, 279–288. doi: 10.1111/mbe.12359

[ref9003] Bellec P. Lavoie-Courchesne S. Dickinson P. Lerch J. P. Zijdenbos A. P. Evans A. C. (2012). The pipeline system for Octave and Matlab (PSOM): a lightweight scripting framework and execution engine for scientific workflows. Front Neuroinform. 6:7. doi: 10.3389/fninf.2012.00007 22493575 PMC3318188

[ref4] Bowden E. M. Jung-Beeman M. (2003). Normative data for 144 compound remote associate problems. Behav. Res. Methods Instrum. Comput. 35, 634–639. doi: 10.3758/BF03195543, 14748508

[ref5] Bullmore E. Sporns O. (2009). Complex brain networks: graph theoretical analysis of structural and functional systems. Nat. Rev. Neurosci. 10, 186–198. doi: 10.1038/nrn2575, 19190637

[ref6] Cassotti M. Agogué M. Camarda A. Houdé O. Borst G. (2016). Inhibitory control as a Core process of creative problem solving and idea generation from childhood to adulthood. New Dir. Child Adolesc. Dev. 2016, 61–72. doi: 10.1002/cad.20153, 26994725

[ref7] Chang Y. L. Wu J. Y. Chen H. C. Wu C. L. (2016). The development of Chinese radical remote associates test. Psychol. Test. 63, 59–81. doi: 10.7108/PT.201603_63(1).0003

[ref8] Chein J. M. Weisberg R. W. (2014). Working memory and insight in verbal problems: analysis of compound remote associates. Mem. Cogn. 42, 67–83. doi: 10.3758/s13421-013-0343-4, 23864281

[ref9001] Chen Y. T. vanEde F. Kuo B. C. (2022). Alpha Oscillations Track Content-Specific Working Memory Capacity. J Neurosci Offic J Societyr Neurosci. 42, 7285–7293. doi: 10.1523/JNEUROSCI.2296-21.2022 PMC9512572 35995565

[ref9] Cui Z. Zhong S. Xu P. He Y. Gong G. (2013). PANDA: a pipeline toolbox for Analyzing brain diffusion images. Front. Hum. Neurosci. 7, 7–42. doi: 10.3389/fnhum.2013.00042 23439846 PMC3578208

[ref10] Dajani D. R. Uddin L. Q. (2015). Demystifying cognitive flexibility: implications for clinical and developmental neuroscience. Trends Neurosci. 38, 571–578. doi: 10.1016/j.tins.2015.07.003, 26343956 PMC5414037

[ref12] Diamond A. (2013). Executive functions. Annu. Rev. Psychol. 64, 135–168. doi: 10.1146/annurev-psych-113011-143750, 23020641 PMC4084861

[ref13] Doss M. K. Považan M. Rosenberg M. D. Sepeda N. D. Davis A. K. Finan P. H. . (2021). Psilocybin therapy increases cognitive and neural flexibility in patients with major depressive disorder. Transl. Psychiatry 11:574. doi: 10.1038/s41398-021-01706-y, 34750350 PMC8575795

[ref14] El Haj M. (2016). Memory suppression in Alzheimer's disease. Neurol. Sci. 37, 337–343. doi: 10.1007/s10072-015-2441-5 26700801

[ref15] Ellis D. M. Ball B. H. Kimpton N. Brewer G. A. (2020). The role of working memory capacity in analytic and multiply-constrained problem-solving in demanding situations. Q. J. Exp. Psychol. 73, 920–928. doi: 10.1177/1747021820909703, 32052699

[ref16] Funch Uhre V. Melissa Larsen K. Marc Herz D. Baaré W. Katrine Pagsberg A. Roman Siebner H. (2022). Inhibitory control in obsessive compulsive disorder: a systematic review and activation likelihood estimation meta-analysis of functional magnetic resonance imaging studies. NeuroImage. Clin. 36:103268. doi: 10.1016/j.nicl.2022.103268, 36451370 PMC9723317

[ref17] Gavazzi G. Noferini C. Benedetti V. Cotugno M. Giovannelli F. Caldara R. . (2023). Cultural differences in inhibitory control: an ALE Meta-analysis. Brain Sci. 13:907. doi: 10.3390/brainsci13060907, 37371385 PMC10295933

[ref19] Gurrieri R. Gambini M. Pescini E. Mastrogiacomo D. Russomanno G. Marazziti D. (2025). Memory functions in obsessive-compulsive disorder. Brain Sci. 15:492. doi: 10.3390/brainsci15050492, 40426663 PMC12110489

[ref20] Horowitz-Kraus T. Randell K. Morag I. (2023). Neurobiological perspective on the development of executive functions. Acta Paediatrica (Oslo, Norway: 1992) 112, 1860–1864. doi: 10.1111/apa.16883, 37338188

[ref21] Hsu C. C. Chen H. C. Chiu F. C. (2012). The development of unusual uses of the newspapers test. J. Chin. Creativ. 3, 33–56. doi: 10.30081/CESJ.201209.000

[ref22] Hu S. Ide J. S. Zhang S. Li C. R. (2016). The right superior frontal gyrus and individual variation in proactive control of impulsive response. J. Neurosci. Off. J. Soc. Neurosci. 36, 12688–12696. doi: 10.1523/JNEUROSCI.1175-16.2016, 27974616 PMC5157110

[ref23] Huang P.-S. Liu C.-H. Chen H.-C. (2019). Examining the applicability of representational change theory for remote associates problem solving with eye movement evidence. Think. Skills Creat. 31, 198–208. doi: 10.1016/j.tsc.2018.12.001

[ref24] Javaid S.-F. Pandarakalam J.-P. (2021). The association of creativity with divergent and convergent thinking. Psychiatr. Danub. 33, 133–139. doi: 10.24869/psyd.2021.133, 34185732

[ref25] Kenett Y. N. Faust M. (2019). A semantic network cartography of the creative mind. Trends Cogn. Sci. 23, 271–274. doi: 10.1016/j.tics.2019.01.007, 30803872

[ref9002] Khalil R. Godde B. Karim A. A. (2019). The Link Between Creativity, Cognition, and Creative Drives and Underlying Neural Mechanisms. Front Neural Circu. 13:18. doi: 10.3389/fncir.2019.00018 PMC6440443 30967763

[ref9005] Khalil R. Karim A. A. Kondinska A. Godde B. (2020). Effects of transcranial direct current stimulation of left and right inferior frontal gyrus on creative divergent thinking are moderated by changes in inhibition control. Brain Struc Func. 225, 1691–1704. doi: 10.1007/s00429-020-02081-y PMC7321900 32556475

[ref26] Kobayashi Y. Morizumi T. Nagamatsu K. Shimizu Y. Kamiya K. Sasaki T. . (2021). Persistent working memory impairment associated with cerebral infarction in the anterior cingulate cortex: a case report and a literature review. Int. Med. (Tokyo, Japan) 60, 3473–3476. doi: 10.2169/internalmedicine.6927-20, 33994436 PMC8627815

[ref27] Koechlin E. (2016). Prefrontal executive function and adaptive behavior in complex environments. Curr. Opin. Neurobiol. 37, 1–6. doi: 10.1016/j.conb.2015.11.004, 26687618

[ref28] Lee C. S. Huggins A. C. Therriault D. J. (2014). A measure of creativity or intelligence? Examining internal and external structure validity evidence of the remote associates test. Psychol. Aesthet. Creat. Arts 8, 446–460. doi: 10.1037/a0036773

[ref29] Leemans A. Jones D. K. (2009). The B-matrix must be rotated when correcting for subject motion in DTI data. Magn. Reson. Med. 61, 1336–1349. doi: 10.1002/mrm.21890, 19319973

[ref31] Liao X. Yuan L. Zhao T. Dai Z. Shu N. Xia M. (2015). Spontaneous functional network dynamics and associated structural substrates in the human brain. Front. Hum. Neurosci. 9:478. doi: 10.3389/fnhum.2015.00478, 26388757 PMC4559598

[ref32] Mansouri F. A. Tanaka K. Buckley M. J. (2009). Conflict-induced behavioural adjustment: a clue to the executive functions of the prefrontal cortex. Nat. Rev. Neurosci. 10, 141–152. doi: 10.1038/nrn2538, 19153577

[ref34] Mattavelli G. Gorrino I. Tornaghi D. Canessa N. (2024). Cognitive and motor impulsivity in the healthy brain, and implications for eating disorders and obesity: a coordinate-based meta-analysis and systematic review. Cortex 171, 90–112. doi: 10.1016/j.cortex.2023.10.008, 37984247

[ref38] Michinov N. Jamet E. Métayer N. Le Hénaff B. (2015). The eyes of creativity: impact of social comparison and individual creativity on performance and attention to others’ ideas during electronic brainstorming. Comput. Hum. Behav. 42, 57–67. doi: 10.1016/j.chb.2014.04.037

[ref39] Miyake A. Friedman N. P. Emerson M. J. Witzki A. H. Howerter A. Wager T. D. (2000). The unity and diversity of executive functions and their contributions to complex "frontal lobe" tasks: a latent variable analysis. Cogn. Psychol. 41, 49–100. doi: 10.1006/cogp.1999.0734, 10945922

[ref40] Morie K. P. Potenza M. N. (2021). A Mini-review of relationships between Cannabis use and neural foundations of reward processing, inhibitory control and working memory. Front. Psych. 12:657371. doi: 10.3389/fpsyt.2021.657371, 33967859 PMC8100188

[ref41] Radel R. Davranche K. Fournier M. Dietrich A. (2015). The role of (dis)inhibition in creativity: decreased inhibition improves idea generation. Cognition 134, 110–120. doi: 10.1016/j.cognition.2014.09.001, 25460384

[ref43] Roger E. Banjac S. Thiebaut de Schotten M. Baciu M. (2022). Missing links: the functional unification of language and memory (L∪M). Neurosci. Biobehav. Rev. 133:104489. doi: 10.1016/j.neubiorev.2021.12.012, 34929226

[ref44] Saldarini F. Gottlieb N. Stokes P. R. A. (2022). Neural correlates of working memory function in euthymic people with bipolar disorder compared to healthy controls: a systematic review and meta-analysis. J. Affect. Disord. 297, 610–622. doi: 10.1016/j.jad.2021.10.084, 34715175

[ref45] Schaefer A. Kong R. Gordon E. M. Laumann T. O. Zuo X.-N. Holmes A. J. . (2018). Local-global parcellation of the human cerebral cortex from intrinsic functional connectivity MRI. Cereb. Cortex 28, 3095–3114. doi: 10.1093/cercor/bhx179, 28981612 PMC6095216

[ref46] Seamans J. K. Floresco S. B. (2022). Event-based control of autonomic and emotional states by the anterior cingulate cortex. Neurosci. Biobehav. Rev. 133:104503. doi: 10.1016/j.neubiorev.2021.12.026, 34922986

[ref47] Smith S. M. Jenkinson M. Woolrich M. W. Beckmann C. F. Behrens T. E. Johansen-Berg H. . (2004). Advances in functional and structural MR image analysis and implementation as FSL. NeuroImage 23, 208–219. doi: 10.1016/j.neuroimage.2004.07.051 15501092

[ref48] Swainson R. Cunnington R. Jackson G. M. Rorden C. Peters A. M. Morris P. G. . (2003). Cognitive control mechanisms revealed by ERP and fMRI: evidence from repeated task-switching. J. Cogn. Neurosci. 15, 785–799. doi: 10.1162/089892903322370717, 14511532

[ref49] Tian H. Wang Z. Meng Y. Geng L. Lian H. Shi Z. . (2025). Neural mechanisms underlying cognitive impairment in depression and cognitive benefits of exercise intervention. Behav. Brain Res. 476:115218. doi: 10.1016/j.bbr.2024.115218, 39182624

[ref50] Torrance E. P. (1974). The Torrance Tests of Creative Thinking: Norms-Technical Manual. Personal Press: Princeton.

[ref51] Tzourio-Mazoyer N. Landeau B. Papathanassiou D. Crivello F. Etard O. Delcroix N. . (2002). Automated anatomical labeling of activations in SPM using a macroscopic anatomical parcellation of the MNI MRI single-subject brain. NeuroImage 15, 273–289. doi: 10.1006/nimg.2001.0978, 11771995

[ref52] Vartanian O. Navarrete G. Chatterjee A. Fich L. B. Leder H. Modroño C. . (2013). Impact of contour on aesthetic judgments and approach-avoidance decisions in architecture. Proc. Natl. Acad. Sci. USA 110, 10446–10453. doi: 10.1073/pnas.1301227110 23754408 PMC3690611

[ref53] Wang R. Benner T. Sorensen A. G. Wedeen V. J. (2007). Diffusion toolkit: a software package for diffusion imaging data processing and tractography. Proc. Intl Soc. Mag. Reson. Med 15:3720.

[ref54] Wang J. Sakata C. Moriguchi Y. (2021). The neurobehavioral relationship between executive function and creativity during early childhood. Dev. Psychobiol. 63:22191. doi: 10.1002/dev.22191, 34674250

[ref55] Wang M. Yu J. Kim H. D. Cruz A. B. (2025). Neural correlates of executive function and attention in children with ADHD: an ALE meta-analysis of task-based functional connectivity studies. Psychiatry Res. 345:116338. doi: 10.1016/j.psychres.2024.116338, 39947841

[ref58] Wu C.-L. (2019). Discriminating the measurement attributes of the three versions of Chinese remote associates test. Think. Skills Creat. 33:100586. doi: 10.1016/j.tsc.2019.100586

[ref59] Wu C.-L. (2022). How one plus one is more than two? The interaction between two players in online co-creativity tasks. Educ. Technol. Soc. 25, 60–70. doi: 10.30191/ETS.202207_25(3).0005

[ref60] Wu C.-L. (2023). The moderating effect of the DMN connectivity on the correlation between online creativity performances in single- and paired-player modes. Intelligence 100:101785. doi: 10.1016/j.intell.2023.101785

[ref61] Wu C.-L. (2024). The moderating effects of brain network connectivity on the relationship between individual and interactive creativity. Think. Skills Creat. 53:101625. doi: 10.1016/j.tsc.2024.101625

[ref62] Wu C.-L. Chen P.-Z. (2022). Differences in online creative performance of single and paired players in cooperative and competitive task situations. Thinking Skills and Creativity, 44, 1–9. doi: 10.1016/j.tsc.2022.101037

[ref63] Wu C. C. Chen F. Y. Kuo C. T. Lin W. W. Liu T. H. Chen Y. H. (1998). Development of revised creative thinking test. J. Stud. Counsel. 62, 132–147.

[ref64] Wu H. Y. Kuo B. C. Huang C. M. Tsai P. J. Hsu A. L. Hsu L. M. . (2020). Think hard or think smart: network reconfigurations after divergent thinking associate with creativity performance. Front. Hum. Neurosci. 14:571118. doi: 10.3389/fnhum.2020.571118, 33328929 PMC7714934

[ref65] Wu C.-L. Su Y.-D. Chen E. Chen P.-Z. Chang Y.-L. Chen H.-C. (2022). Development and validation of interactive creativity task platform. Front. Psychol. 13:954946. doi: 10.3389/fpsyg.2022.954946, 35992391 PMC9386566

[ref66] Yalçın V. Erden S. (2021). The effect of STEM activities prepared according to the design thinking model on preschool children’s creativity and problem-solving skills. Think. Skills Creat. 41:100864. doi: 10.1016/j.tsc.2021.100864

[ref67] Zabelina D. L. Friedman N. P. Andrews-Hanna J. (2019). Unity and diversity of executive functions in creativity. Conscious. Cogn. 68, 47–56. doi: 10.1016/j.concog.2018.12.005, 30634127 PMC6594373

[ref68] Zalesky A. Fornito A. Harding I. H. Cocchi L. Yücel M. Pantelis C. . (2010). Whole-brain anatomical networks: does the choice of nodes matter? NeuroImage 50, 970–983. doi: 10.1016/j.neuroimage.2009.12.027, 20035887

[ref70] Zhao X. Zhang W. Tong D. Maes J. H. R. (2023). Creative thinking and executive functions: Associations and training effects in adolescents. Psychology of Aesthetics, Creativity, and the Arts, 17, 79–90. doi: 10.1037/aca0000392

